# Enhancing the Facilitation of Interprofessional Education Programs: An Institutional Ethnography

**DOI:** 10.3390/nursrep11030052

**Published:** 2021-07-16

**Authors:** Nadine Ezzeddine, Sheri Lynn Price

**Affiliations:** School of Nursing, Dalhousie University, Halifax, NS B3H 4R2, Canada; sheri.price@dal.ca

**Keywords:** interprofessional education, IPE facilitation, institutional ethnography, stereotypes

## Abstract

Interprofessional collaboration (IPC) among health care professionals has been identified as essential to enhance patient care. Interprofessional education (IPE) is a key strategy towards promoting IPC. Several factors including the nature of facilitation shape the IPE experience and outcomes for students. Stereotypes held by students have been recognized as a challenge for IPE and IPC. This study aimed to explore institutional rules and regulations that shape facilitators’ work in IPE interactions problematized by students’ stereotypes at a university in Atlantic Canada. Employing institutional ethnography as a method of investigation, data were collected through observations, interviews, focus groups, and written texts (such as course syllabi). Participants included three facilitators, two undergraduate nursing students, and two IPE committee members of an IPE program. Findings revealed four work processes conducted by facilitators in local IPE settings related to students’ stereotypes. These processes were shaped by translocal discourse and included the work used to form teams, facilitate student introductions to team members, facilitate team dynamics, and provide course content and context. Study results included the identification of several strategies to address student stereotypes and enhance collaboration, including directions for future curriculum decisions and the pedagogical organization of IPE.

## 1. Introduction

Interprofessional collaboration (IPC) among health care professionals has been identified as essential to promote safe patient care and is a key consideration in health human resources planning [1]. To enhance IPC, interprofessional education (IPE) was introduced into health professions educational programs. IPE “occurs when two or more professions learn about, from and with each other to enable effective collaboration and improve health outcomes” [1] (p. 13). 

The design of several IPE curricula has been guided by competency frameworks to promote collaborative competencies such as the Canadian Interprofessional Health Collaborative (CIHC) Interprofessional Competency Framework in Canada [2]. The CIHC framework provides six competencies: role clarification, patient/client/family/community-centered care, team functioning, collaborative leadership, interprofessional communication, and interprofessional conflict resolution [2]. The CIHC framework has been adopted at the IPE program this study investigated. 

Stereotypes held by health care profession students have been described as a challenge for the development, delivery, and effectiveness of IPE programs. Stereotypes are defined as beliefs assumed about an individual based on whatever is generally known about the social or professional group that individual belongs to [3]. Students might hold stereotypes about their own profession and/or another health profession [4]. Stereotypes can have positive outcomes, but often have negative outcomes when they involve inaccurate beliefs about members of the same or different groups [4]. 

Research examining stereotypes among health care profession students revealed that health care students join their programs with pre-established stereotypes. Survey studies showed that pre-entry students perceived physicians as having the highest academic ability, strong leadership, and decision making, whereas nurses were perceived as team players, with practical and interpersonal skills [5], and lacking the ability to work independently [6]. Nurses and registered dietitians were perceived to have fewer leadership skills [6].

The existing research evaluating the ability of IPE programs to ameliorate the stereotypes students hold is inconclusive and mostly quantitative. Survey studies described an ameliorating effect of IPE on student stereotypes; a study by Ateah et al. [7] showed that health profession students initially rated the physicians highest for independence and nurses lowest, and after IPE experience, all professions achieved equal rating on this attribute. Other studies showed that IPE programs were not successful in changing students’ stereotypes; a study by Hawkes et al. [8] revealed that health profession students viewed medicine as least caring both before and after an IPE experience, and that all students perceived their own profession as more caring both before and after the IPE experience. Yet, other studies described an exaggeration of student stereotypes after IPE programs. Most participants in Leaviss’ [9] study reported developing negative attitudes towards medical students, and occupational therapists and physiotherapists reported developing negative attitudes towards each other. 

One of the researchers in this study, who is also an IPE facilitator at a university in Atlantic Canada (the setting of this study), identified and experienced the existence of stereotypes among students. Knowing that course organizers and facilitators at this IPE program adopted the CIHC framework (including role clarification competency) to guide the preparation and delivery of IPE courses, the researchers identified the existence of students’ stereotypes as an area of concern. 

The researchers employed an institutional ethnographic design to (a) identify the work processes performed by IPE facilitators that are related to the area of concern in this study—student stereotypes, and (b) explicate the institutional rules, regulations, and limits that determined these work processes within an IPE program at a university in Atlantic Canada.

## 2. Materials and Methods

### 2.1. Method of Investigation

Institutional ethnography (IE) was developed by the contemporary sociologist Dorothy Smith [10]. IE aids to understand the experiences of people in institutions. IE helps in ‘knowing the known’ and ‘understanding the understood’ by uncovering the underlying relations and ideas that are not obvious [11]. IE allows researchers to examine how institutions’ rules and regulations (such as IPE course syllabi) determine the work experiences of those in the institution (IPE facilitators) and impact the daily lives of all involved (facilitators and students) [10,11]. 

The investigation in institutional ethnography involves two settings: (1) the local setting, such as an IPE classroom where facilitators and students indulge in their IPE experiences as stated by the syllabus, and (2) the translocal, an outside setting where rules and policies are determined remotely and crafted in texts, such as course organizers and ruling committees (translocal setting) determining the course syllabi and IPE policies that dictate what happens in IPE class [10]. 

Texts are “words, images, sounds that are set into a material form of some kind from which they are read, seen, heard, watched, and so on” [10] (p.66). Texts (as IPE course syllabi) are prepared translocally informed by the interests and ideologies of people at the translocal setting. Once these texts are activated by people at local sites, these concepts become common discourse [10]. 

Internal institutional discourse refers to the common institutional texts, rules and regulations that dictate the work that IPE facilitators perform across different IPE courses. Institutional ethnography calls for considering discourses that do not comply with internal institutional discourses because of “extra-institutional talk” and “oppositions critical talk” [10]. Extra-institutional talk refers to discourse structured by other agencies: students in a local IPE setting interact with other profession students based on observations in hospital settings (other agency) that reinforce the role of physicians as ultimate decision makers. “Oppositions critical talk” highlights what an individual facilitator might do differently than the common institutional discourse (implemented by facilitators across IPE courses). The focus was to discover how and which discourse operated in these” extra-institutional talk” and “oppositions critical talk” and what difference it made for students and facilitators.

An IE study typically involves (1) two stages of data collection, (2) ongoing data analysis, and (3) mapping the findings [11]. The first stage of data collection aims to identify the area of concern to be explicated. As mentioned earlier, the area of concern was the stereotypes held by students about their own and other’s profession.

### 2.2. Setting

This study explored an IPE program at a university in Atlantic Canada. At the time of this study, the program provided IPE learning activities for approximately 3000 students from Medicine, Dentistry and undergraduate health professions programs. These students were required to participate in different IPE activities throughout their programs. This study focused on exploring the work processes used by faculty in relation to the teaching methods of face-to-face instructions and simulations.

### 2.3. Participants

Institutional ethnography does not identify the typical sample size; the sample size corresponds to the number of participants needed to investigate the work performed. Participants in this study included two nursing students; three IPE facilitators (two of which were both IPE facilitators and course organizers); and two representatives of the IPE coordinating committee (who also hold the position of IPE coordinator in their own faculty/school). 

IPE facilitators are faculty members of either the Faculty of Health, Dentistry, or Medicine who had participated (at the time of this study) in the development and/or implementation of one or more IPE experience in the same university. 

Nursing students were selected given the researchers’ positionality as nurses. The initial sample size targeted was 6 nursing students, a size recognized as appropriate for a focus group interview [12], but only two nursing students were recruited from a cohort of approximately 100 students after three recruitment attempts. One explanation for the low response rate might be the timing; recruitment took place during the second half of the Winter Semester when students were in their clinical placements before their final exams. Nevertheless, a sample size of two students did not influence the rigor of this study because it is the facilitators’ work that this study was investigating. The interest in students was to explore the interplay of the facilitators’ work being explored with students’ stereotypes (the area of concern in this study), and as such, any students’ experience is valid.

All participants were recruited through email communication. Emails to nursing students were sent by the undergraduate nursing program, and those for facilitators and committee representatives were sent by the IPE program main office.

### 2.4. Data Collection

Data were collected through observations, interviews, focus groups, and texts [11]. In institutional ethnography, the researchers are required to be immersed in the everyday world of the participants. As such, the main researcher enrolled as a student in eight IPE activities and prepared an IPE course, and the second researcher held a role as an IPE course organizer and facilitator. Reflections and field notes were kept.

One-on-one interviews were conducted with the three facilitator participants (two were also course organizers) and the two IPE coordinating committee representatives (who were also course organizers). Interviews were semi-structured, took place in closed private settings, were recorded, and lasted 90–120 min. 

Two types of focus group interviews were conducted; each was recorded and lasted 90 min. The first focus group was composed of the two nursing students, who discussed how their IPE experiences shaped the development and sustainability of their stereotypes. The second focus group interview included one IPE facilitator participant and the two nursing students (from the first focus group) who reflected on the discussions held in the first focus group. The second focus group was conducted to achieve reflexivity which is one way of ensuring trustworthiness in IE.

Texts analyzed included course syllabi, course material and cases, frameworks and models, and the facilitators’ guide.

### 2.5. Data Analysis

Data analysis involved identifying work processes taking place in local IPE settings, interfaces of work processes (between local and translocal settings), institutional discourse analysis, and mapping [11].

Work processes occurring at local settings were identified and explicated by analyzing data collected through observations, reflections, field notes, and reading through the interviews and answering a set of questions identified for IE by McCoy [13]. Some of these questions were:

- “What is the work that these informants are describing?
- What does it involve for them?
- How is their work connected with the work of other people?
- What particular skills or knowledge seems to be required?
- What does it feel like to do this work? and
- How is the work articulated to institutional work processes?” ([13], p. 111)

The second step focused on interfaces of work processes to identify the translocal setting and the specific work processes related to student stereotypes. By reading through the interviews, reflections, field notes, and memos, the key question asked was “Why the work takes this form?” [13].

The next step of analysis involved identifying and analyzing texts [13] to identify and explicate the internal institutional discourse, the “extra-institutional talk” and the “oppositions critical talk” (explained earlier). 

The final step of analysis was to draw maps to show the relation between each work with a specific text prepared in a translocal setting. Turner [14] presented the following instructions for drawing maps:

- Circles indicate the work activity performed by facilitators who use a text.
- Boxes indicate the texts that are prepared translocally and shape local work practices.
- Solid lines indicate that the text is also available in local settings.
- Dotted lines indicate that the text in not available in local settings.

## 3. Results

Our findings revealed four work processes conducted by facilitators in local IPE settings related to the area of concern in this study (students’ stereotypes). These included the work used: (1) in the formation of teams, (2) to facilitate student introductions to team members, (3) to facilitate team dynamics, and (4) to provide course content.

### 3.1. Forming Teams

**Figure 1:**
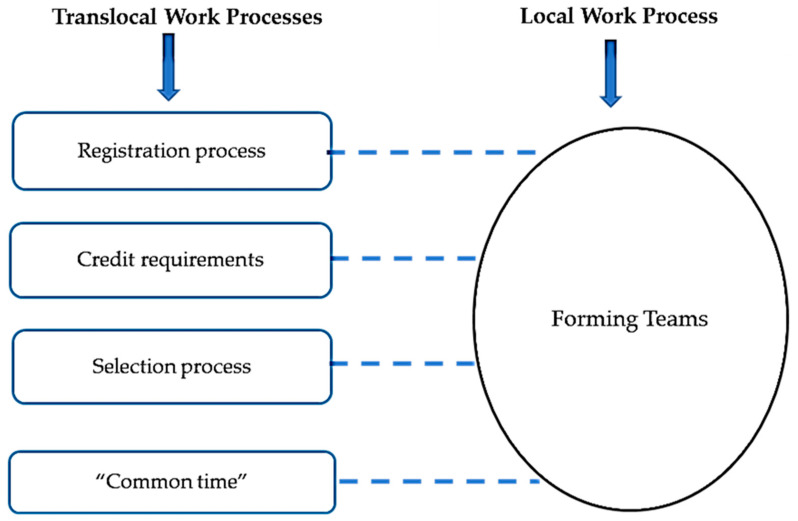
Forming Teams work process.

In a local IPE setting, in the first session, each facilitator would subdivide enrolled students into working teams that incorporate diversity in the professions represented, number of students representing each profession, and students’ program year. Figure 1 maps how this local work process was determined by the following translocal discourses: (1) the Faculty of Health (FH) courses registration process; (2) the FH courses scheduling process; and (3) the course organizers’ selection process. The Faculty of Health (FH) courses registration process is based on each professions’ program credit requirements and student’s own interest in the IPE course topic. The FH courses scheduling process designated a “common time” (Tuesdays and Thursdays of each week between 3:30 pm and 5:00 pm) for IPE courses, and students could only register if no departmental courses were offered during this time. The third translocal work process was the way course organizers selected students who have registered in their course, which was decided on a “first come first served” basis.

In relation to the area of concern in this study (student’s stereotypes), participants in the local setting (all students plus the one facilitator) identified that a lack of team balance, especially in relation to having first-year and fourth-year students together, was noted to promote and perpetuate the development of stereotypes. Fourth-year students, regardless of their profession, perceived themselves as leaders, which was commonly operationalized by them dominating class discussions. First-year students tended to behave as followers, operationalized by assuming a passive, listening role. One nursing student participant stated.

“There wasn’t really much of interacting… It was more like first year nursing students …receiving information from [profession] students …we all felt underpowered… I thought we were going to work together but … we’re just first years listening to a lecture by them.”

### 3.2. Facilitating Students’ Introductions

**Figure 2:**
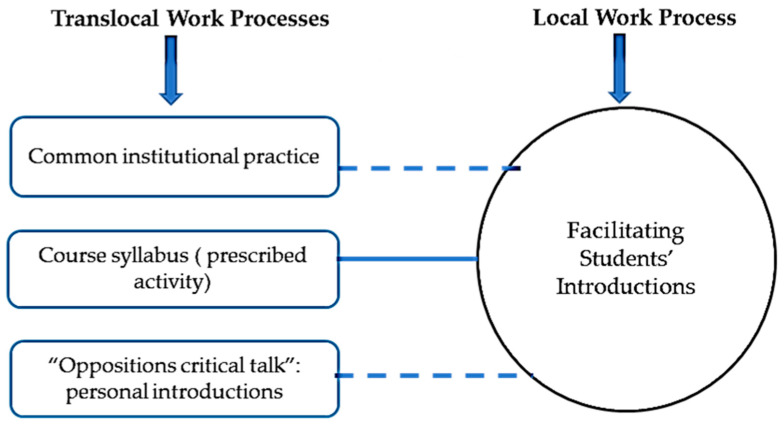
Students’ Introductions work process.

In a local IPE classroom, facilitators structured student introductions at the beginning of the first session to be very brief (5 to 10 min for all introductions), asking each student only to introduce their name and department, which did little to help students know one another in any depth. As depicted in Figure 2, this local work process was shaped by three translocal discourses: (1) a common institutional practice employing the same short introduction process across IPE courses; (2) an IPE activity (among others) with limited time as prescribed by course organizers and communicated to facilitators through the course syllabus and emails, and (3) an “oppositions critical talk” by one IPE course organizer/facilitator participant who emphasized the communication of personal information in the introductions. This participant believed that relating to one another as humans is central for collaboration, and that this cannot be achieved through standard short introductions. This participant shared “If we really want to have good interprofessional collaboration, understanding who we are as humans first and then moving to our professions.”

As a course organizer, this participant introduced a new discourse in her course(s) and revised other planned IPE activities to accommodate longer timeframes for the new personal introduction process. Every student was asked to share some personal information or interest to facilitate students’ understanding of one another as individuals beyond a name and profession, “one of the things I started asking [to students] … is as you introduce yourself… then I want to know your discipline, then I want to know something that is important to you, as a person...” 

In relation to the area of concern, student participants shared that introducing themselves solely in the context of their profession emphasized the profession over the person and only reinforced pre-held stereotypes about that profession. One student participant stated “with all of the different stereotypes… that we already have coming in, … now you see the person as their role and not who they are as an individual.” 

Student participants also expressed an inclination to establish a human connection with other students before discussing professional roles, aligned with the experience shared by the organizer/facilitator. One student participant shared.

“Everybody is a human first regardless of what role you have… to know them as a person—because we all have the same goal... to care for the patient… But if we put the stereotype in front of that, it’s just like a barrier…”

### 3.3. Facilitating Team Dynamics

In local IPE settings that employed simulated interviews as a teaching method, facilitators introduced the expected activities. Each team of different health professions would meet for a short time to plan the interview with a simulated patient and their family/caregivers. Team members then interviewed the simulated patient and caregivers, and every student asked at least one question. This was followed by a face-to-face group debrief. 

**Figure 3:**
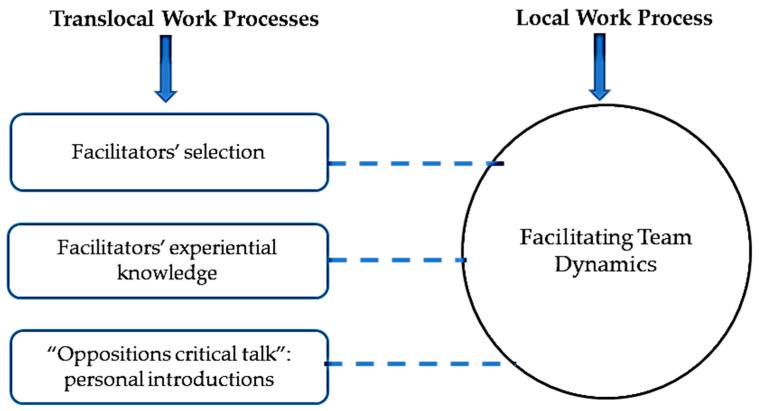
Facilitating Team Dynamics work process.

Facilitating team dynamics involved attending to the students’ and simulated patient’s body language during the interview, ensuring student engagement, and assessing the ease of discussion. If the facilitator identified an issue during the interview, they either intervened on the spot or waited until the group debrief. Figure 3 shows that translocal discourses included (1) the process through which course organizers selected facilitators based primarily on the knowledge of and skill in the course topic (and not necessarily interprofessional competencies); (2) a lack of a formal facilitator training program at the Atlantic university; and (3) the facilitators’ own knowledge of and skills in facilitating team dynamics, typically informed by their own professional experience which may or may not be appropriate for the interprofessional context. One facilitator participant who had the sole role of facilitator shared “I was selected based on a recommendation of a faculty member…we didn’t even have a training session… they [the faculty members] generally work with different team members, but do they actually work collaboratively? I don’t know.” 

Student participants reflected that the way facilitators addressed team dynamics, specifically in conflicts that arose when students’ stereotypes were operationalized, could both reinforce existing stereotypes and introduce new ones. A student participant shared: “[in a group debrief] a team member brought up something said [during a conflict that occurred upon discussing roles] … my facilitator tried to make light of that… but she wasn’t really addressing it…”. This participant also expressed a lack of confidence in the facilitator’s skills and knowledge in addressing such conflicts and team dynamics’ “I still feel not respected… I think they [facilitators] try to [facilitate team dynamics] but they just don’t know how.”

These expressions of embodied experience revealed a feeling of being “not respected” by another profession, a situation that could reinforce existing expressed stereotypes and create new stereotypes (e.g., in the participant towards that profession). 

### 3.4. Delivering Course Content

**Figure 4:**
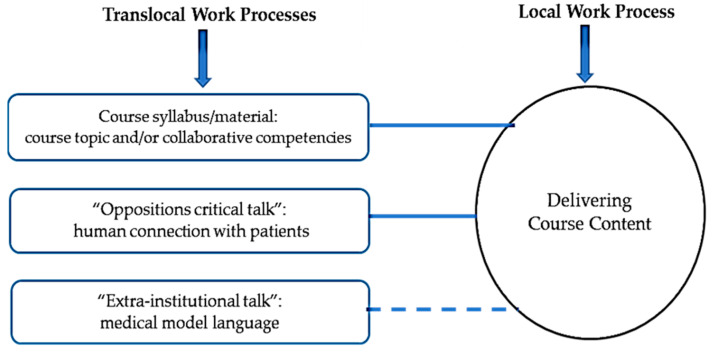
Delivering course content work process.

In local IPE courses, and in the first session, facilitators shared with the students the course objectives, syllabus, guiding framework(s) and tools. In subsequent class sessions, facilitators might share new material, which included the case of a simulated patient in simulated courses. The content and objectives varied between courses; for example, the objectives in some courses focused on the topic of the courses, while the focus was on the CIHC collaborative competencies in others. As illustrated in Figure 4, this process of course objectives selection was shaped translocally by (1) the course organizers’ knowledge of and expertise in the course topic, (2) the organizers’ personal interest and agendas in providing an IPE course in a specific topic, (3) the collaborative competencies outlined in the CIHC, (iv) “oppositions critical talk” promoting humanistic communication, and (4) “extra-institutional talk” emphasizing the dominance of the medical model.

Even though the IPE program studied adopted the CIHC collaborative competency framework, the focus of courses varied and at times did not include any of the collaborative competencies. IPE departmental coordinators clarified that it is the course organizers who decide the content, and that the focus might at times be based on their own interest and goals. Another IPE coordinator opined that facilitator’s/organizer’s lack of expertise and knowledge about collaborative competencies was a major factor for organizers’ focus on a specific topic rather than on collaborative competencies. Translocal discourse also included an “oppositions critical talk”, where one organizer/facilitator reinforced establishing therapeutic communication and rapport with patients which she believed to be a pre-requisite for patient-centered care as a collaborative competency. This organizer/facilitator employed Egan and Schroeder’s [15] “establishing therapeutic communication” theory and focused on a social topic that serves as a context for establishing connection with patients, and explained

“[the student] was using something that she understood quite well... outside her discipline, but it helped connect her with the patient… the person felt connected. So, recognizing that we are humans first, then we have a discipline, and then we are working with you as a team.”

Translocal “extra-institutional talk” revealed that the inherent medical model of health care settings functioned as a text shaping what takes place in educational and IPE settings. The participant with the sole role of facilitator shared

“The curriculum that we are learning, it’s a medical curriculum... we start from the diagnosis, then we make all our plans as health professions…we are not starting from something humane… what about nursing here… what about the social work. So, it is all based on the diagnosis.”

Student participants described how the education and simulation exercises they experienced in IPE activities reinforced the dominance of physicians. One student participant shared “the student physician wanted to order a drug… I heard him verbally say the order but... I felt like a subordinate who wanted him to write his signature…just to cover myself.”

Students emphasized the power of the medical model in shaping stereotypes, clinical practices, and even the education provided in the IPE program. They considered that leadership roles were embedded within scopes of practice that generally reinforce the role of physicians as leaders who write orders for other health professions to follow. Students admitted that they entered their undergraduate educational programs with pre-existing stereotypes, and that, as IPE courses generally lacked objectives and content that directly addressed stereotypes, they did not necessarily help in ameliorating their stereotypes. As one student participant shared “there will always be a stereotype… we will always follow physician’s orders... that is their scope of practice… And that’s our scope of practice”.

## 4. Discussion

This study identified the following four work processes conducted by facilitators in local IPE settings and related to students’ stereotypes: (1) forming teams, (2) facilitating team introductions, (3) facilitating team dynamics, and (4) providing course content. The regulations determining these work processes revealed institutional limits imposed on facilitators that would impede their ability to promote students’ learning with, from, and about each other and hence shape students’ stereotypes. These work processes were dictated by translocal discourses such as the “first come first-served” student selection process, the common institutional practice of short student introductions, the lack of formal training for IPE facilitators, and the adoption of the CIHC framework to guide IPE activities. Facilitators had no say in or control over these work processes unless they were also course organizers. This study uncovered several such translocal discourses, each of which represents an opportunity for change and improvement. The findings also revealed new translocal discourses introduced by specific facilitators/course organizers as “critical-oppositions talk” which aimed to re-shape the local work processes and hence ameliorate the area of concern—student-held stereotypes. 

Our findings emphasize the importance of establishing personal and human connections among students and with patients as a discourse for acquiring collaborative competencies and ameliorating student-held stereotypes. Whereas this is a new finding that the published IPE literature does not specifically address, this article presents Tajfel’s [16] Social Psychology of Intergroup Relations as a supportive theory. Tajfel considered intergroup relations (between different professions) as an operationalization of ethnocentric beliefs. Ethnocentrism occurs when individuals of one group evaluate individuals of another group based on the preconceptions they hold about the second group and which are a result of applying one’s own professional standards to evaluate the second group [16]. It is this dynamic that the call for early personal connections prior to introduction of professions is trying to avoid. Holding an identity of human social beings versus disparate professions can limit the early operationalization of ethnocentric beliefs and stereotypes. 

Our findings also showed that adopting a more personal introduction process can facilitate an environment where members identify, interact, and communicate as social human beings. This can in turn help to initiate an effective process for communication among team members, which is a core collaborative competency [2]. Whereas searching the IPE literature did not yield any evidence about the role of establishing personal connection in introductions on IPE, the evidence from sociology emphasizes the importance of first day introductions in setting the social norms of the class [17]. 

Participants suggested a new translocal discourse for adopting a people-centered approach during the development and delivery of the IPE courses, which could be incorporated through objectives addressing the collaborative competencies of communication and patient-centered care. Guided by the “Therapeutic Relationships” theory by Egan and Schroeder [15], participants proposed that the first IPE activities for students in their first year could be any topic of human interest focusing on communication and patient centeredness. As students progress in their programs and in IPE activities, other collaborative competencies [2] or clinical issues can be integrated into course objectives while maintaining communication and patient-centered care as core competencies.

The findings also revealed that the IPE program studied lacked a scientific IPE facilitator recruitment process and a formal faculty training program. Even though universities across Canada have implemented different IPE faculty development programs, there is limited evidence that supports the effectiveness of these initiatives [18]. Reeves et al. [19] emphasized the importance of proper recruitment, preparation and initial training for new facilitators, and ongoing support for IPE facilitators. Buring et al. [20] argued that IPE faculty development programs should enable facilitators, representing diverse health professions, to become competent in active learning methods, skilled in facilitating group dynamics, and knowledgeable about the roles of the various professions—preferably, prior to serving a facilitator role. 

Our findings also revealed that the dominance of medical model language and practice shaped the area of concern in this study—stereotypes. This discourse may be difficult to address, but there is a need to understand what collaborative practice looks like within the context of the medical model, specifically in promoting leadership and decision making among all team members. The current language within the IPE course content reinforces the dominance and hegemony of the medical model. The term “physician’s order” is a discourse that is worthy of reconsidering to incorporate less hegemonic terms for example. Changing the language may not necessarily change the culture and practice. However, hidden practices, texts and discourses can reinforce negative stereotypes.

## 5. Limitations 

There are some considerations in relation to the methodology and methods of this study. Institutional ethnography is not intended to show causation or be generalized to other populations. However, this research provides an in-depth, contextualized understanding of the limitations of an IPE program that does not currently exist in the published literature.

Another limitation was the difficulty in recruiting nursing student participants; only two students were recruited despite amendments to the recruitment process and providing compensations. Future studies with more students and facilitators from different health professions is needed.

## 6. Implications

The findings of this study hold several practice and research implications. Further understanding of how stereotypes can be identified and addressed is crucial. This may include (1) starting with quantitative studies through surveys to identify the specific stereotypes health care profession students in an IPE program hold, (2) followed by qualitative studies, such as institutional ethnography, to uncover the institutional norms and regulations that shape each specific stereotype. This approach presents an empowering approach to address stereotypes shaped by institutional norms and limitations. 

Several questions also arise is relation to what is a person-centered approach in IPE and how can it be implemented? What is the impact of establishing human connection among students on IPE and students’ stereotypes? Additionally, questions in respect to IPE faculty development program content, guiding frameworks, methods, and the professions/disciplines essential in structuring IPE faculty development programs are important.

## 7. Conclusions

In addition to reinforcing the need for an IPE facilitator training program, this study presented unique findings including the importance of establishing human connection and a people-centered approach within IPE. As such, these findings have significance for collaboration between decision makers (who set the vision and goals of IPE programs), IPE coordinators and committee members, IPE facilitators, and students.

This study also provided a unique understanding of how institutional discourses are embodied into both facilitators’ work and students’ perceptions and stereotypes. By uncovering some of the institutional determinants of the area of concern in this study, facilitators gained an understanding of the institutional translocal limitations of their work, and suggested changes and improvements in the institutional processes and pedagogical practices.

## Data Availability

The data supporting the results are stored in password-protected folders with the primary researcher of this study.
